# Vacancies in Medical Schools

**Published:** 1847-06

**Authors:** 


ARTICLE V.

### VACANCIES IN MEDICAL SCHOOLS.

Robert Hare, M. D., the eminently distinguished Professor of Chemistry in the University of Pennsylvania, has resigned his chair.

We would be gratified to see the venerable institution from which he has retired, lead off in the great and good work of selecting professors by concours, in filling the vacancy thus occasioned. It would have a powerful influence in determining the policy of other schools, in this respect; teaching them to prefer merit to influential social relations.

John Moorehead, M. D., who has for a long time been connected with and filled different chairs at different times in the Medical College of Ohio, has resigned that of Theory and Practice which he last held.

We would urge upon our friends of Ohio, also, the system of concours.

J. G. Norwood, M. D., has resigned the chair of Materia Medica in the University of St. Louis and accepted an appointment in the corps of geologists sent out by the General Government to survey the mineral lands of the north-west.

The death of Prof. Revere has left the chair of Theory and Practice vacant in the University of the city of New York.

And the chair of Surgery in the medical department of Hampden Sydney College of Virginia is vacated by the death of Professor Warner.

What an impression upon the profession for good would be made could all these vacant places be filled by public trials! What imposing scenes to see the intellectual conflicts between the first minds in the profession! What new life and energy it would diffuse into the great body of the profession for its members to feel that by merit they were all entitled to consideration for the first places and rank! Let the system be adopted, and an era will be marked in our history, of far greater importance than any that has been for the last half century, or is likely to be, without it, for half a century to come.

